# The Ets dominant repressor En/Erm enhances intestinal epithelial tumorigenesis in Apc^Min ^mice

**DOI:** 10.1186/1471-2407-9-197

**Published:** 2009-06-22

**Authors:** Paul Jedlicka, Xiaomei Sui, Arthur Gutierrez-Hartmann

**Affiliations:** 1 Department of Pathology, University of Colorado Denver, Aurora CO, USA; 2 Department of Medicine, University of Colorado Denver, Aurora CO, USA; 3 Department of Biochemistry, University of Colorado Denver, Aurora CO, USA; 4 Department of Molecular Genetics, University of Colorado Denver, Aurora CO, USA

## Abstract

**Background:**

Ets transcription factors have been widely implicated in the control of tumorigenesis, with most studies suggesting tumor-promoting roles. However, few studies have examined Ets tumorigenesis-modifying functions *in vivo *using model genetic systems.

**Methods:**

Using mice expressing a previously characterized Ets dominant repressor transgene in the intestinal epithelium (Villin-En/Erm), we examined the consequences of blocking endogenous Ets-mediated transcriptional activation on tumorigenesis in the Apc^Min ^model of intestinal carcinoma.

**Results:**

En/Erm expression in the intestine, at levels not associated with overt crypt-villus dysmorphogenesis, results in a marked increase in tumor number in Apc^Min ^animals. Moreover, when examined histologically, tumors from En/Erm-expressing animals show a trend toward greater stromal invasiveness. Detailed analysis of crypt-villus homeostasis in these En/Erm transgenic animals suggests increased epithelial turnover as one possible mechanism for the enhanced tumorigenesis.

**Conclusion:**

Our findings provide *in vivo *evidence for a tumor-restricting function of endogenous Ets factors in the intestinal epithelium.

## Background

Members of the Ets transcription family, numbering up to 27 in humans, are widely expressed in developing and mature tissues, and regulate diverse cellular processes [1,2]. Ets factors are also frequently misexpressed in the setting of neoplasia. Many Ets factors become overexpressed in tumors and appear to play tumor-promoting roles, while a limited number, notably the epithelial specific Ets, may perform tumor suppressor functions [1,3-5]. However, to date, most information about Ets functions in tumorigenesis has come from cell culture and animal xenograft models. Indeed, very few studies have examined Ets functions in tumorigenesis *in vivo *using model genetic systems [6-8]. Ets factors are widely expressed in the intestine, and often misexpressed in carcinoma of the colon, but their tumor-modifying roles in intestinal epithelial neoplasia *in vivo *largely remain to be defined [9].

We have previously generated and characterized transgenic mice expressing an Ets dominant repressor (En/Erm) with broad Ets-blocking activity in the small intestinal epithelium [10]. Nearly all members of the Ets transcription factor family are expressed in the mature mammalian intestine, but expression levels vary widely [9,11]. Our previous study characterized the phenotypic consequences of transgene expression at immunohistochemically detectable levels, which resulted in marked disturbance of crypt-villus homeostasis [10]. Additional transgenic lines, expressing En/Erm at levels detectable by RT-PCR but not immunohistochemistry ("low expressors"), did not manifest an overt dysmorphogenic phenotype under normal physiologic conditions, despite the fact that En/Erm is able to block Ets activity at substoichiometric levels *in vitro *[10]. To determine whether this low-level En/Erm expression has phenotypic consequences under pathologic conditions, we tested its effect on intestinal epithelial tumorigenesis in the Apc^Min ^mouse, a well-established model of multiple intestinal neoplasia [12-14].

We find that animals with low-level En/Erm expression develop more than twice as many tumors in the small intestine as non-transgenic Apc^Min ^controls. Interestingly, while these animals do not manifest the overt crypt-villus dysmorphogenesis phenotype under conditions of homeostasis previously described for high-level En/Erm expressors [10], they do show a mild increase in epithelial transit. Thus, the increase in tumor number in the En/Erm animals may in part be due to increased crypt-villus epithelial turnover. Moreover, on histologic analysis, tumors from En/Erm-expressing animals show a trend toward greater stromal invasion. Our studies in a genetic tumor model thus uncover an unexpected role for epithelially expressed Ets factors in the restriction of tumorigenesis in the intestine.

## Methods

### Animals

Villin-En/Erm transgenic animals were generated as previously described [10], and were maintained in an FVB/N background. Apc^Min/+ ^animals were obtained from Jackson Laboratories and were maintained in a C57BL/6J genetic background. Experimental and control animals for the tumor study were both derived from a cross between Villin-En/Erm animals and Apc^Min/+ ^animals. The studies were thus carried out in a hybrid (C57BL/6J × FVB/N) background, as done by others [15]. In order to control for possible confounding effects of the modifier-of-Min locus (Mom1), the major modifier of tumor multiplicity in the Apc^Min ^strain [16], the Mom1 genotype (resistant versus sensitive) was determined, as previously described [15,17], and such analyses indicated that all animals in the study were heterozygous (Mom1^S/R^) for the Mom1 locus. The presence of the Villin-En/Erm transgene and Apc^Min ^mutation were determined by PCR genotyping of tail-biopsy DNA, as previously described ([10]; http://jaxmice.jax.org). Tumor number, size and histology in Apc^Min ^and Apc^Min^;Villin-En/Erm animals were evaluated in H+E-stained sections of the complete length of the small intestine by a pathologist (PJ) blinded to the genotype. All animal work was carried out under protocols approved by the Institutional Animal Care and Use Committee.

### Histology and immunohistochemistry

Animals were euthanized using CO_2 _followed by cervical dislocation. The small intestine was immediately harvested and cut into two to three segments of approximately equal length. Fecal contents were gently expelled, the lumen was injected with fixative (4% paraformaldehyde), and the intestine was rolled concentrically and placed in a histology cassette. Fixation was for 24 hours in 4% paraformaldehyde at 4°C, after which the tissues were placed in 70% ethanol, processed further on a standard histology processor and paraffin-embedded. Sections 4 um thick were stained with hematoxylin and eosin (H+E) or processed further for immunohistochemical staining. For immunohistochemical staining, sections were deparaffinized and rehydrated. Antigen retrieval was performed by incubating the slides in 10 mM sodium citrate buffer, pH 6.0, for 1 hour in a Biocare Medical Decloaker. Endogenous peroxidase activity was blocked by incubation in 3% H_2_O_2 _for 10 minutes. Immunohistochemical staining was performed using the M.O.M. (mouse on mouse) kit (Vector Laboratories), and developed using DAB (Dako or Sigma). Primary antibodies used were mouse anti-smooth muscle actin (Dako, 1:100) and mouse anti-E-cadherin (BD Biosciences, 1:100). Mcm6 and β-catenin immunohistochemical staining, and BrdU labeling and immunohistochemical staining were performed as described previously [10]. All immunohistochemically stained slides were counterstained with hematoxylin, dehydrated, mounted and coverslipped.

### RT-PCR

Following euthanasia, the small intestine was removed, cut into multiple segments and fecal contents were gently expelled. The intestinal segments were opened lengthwise and stored in RNAlater (Ambion) at 4°C overnight. The tissue was removed from RNAlater and laid mucosal surface up onto Petri dish lids. The mucosa was gently scraped off with a razor blade and collected in a 1.5 ml tube. Trizol (1 ml; Invitrogen) was added, the tissue was homogenized with a disposable pestle (Fisher), and RNA was isolated per manufacturer protocol (Invitrogen) and stored at -80°C. Ten (10) ug of RNA were treated with DNase using a DNA-free kit (Ambion), and 1.6 ug of treated RNA were reverse transcribed using Superscript III reverse transcriptase (Invitrogen), per manufacturer protocol, using random primers in a 20 ul reaction. In parallel control reactions, reverse transcriptase was omitted. One ul of the reverse transcription reaction was then PCR-amplified using primers to Engrailed (for detection of the transgenic transcript) or Actin (control), as previously described [10], and the products were resolved on a 2% agarose gel stained with ethidium bromide.

## Results

### Intestinal En/Erm expression increases tumor number in Apc^Min ^mice

**Figure 1:**
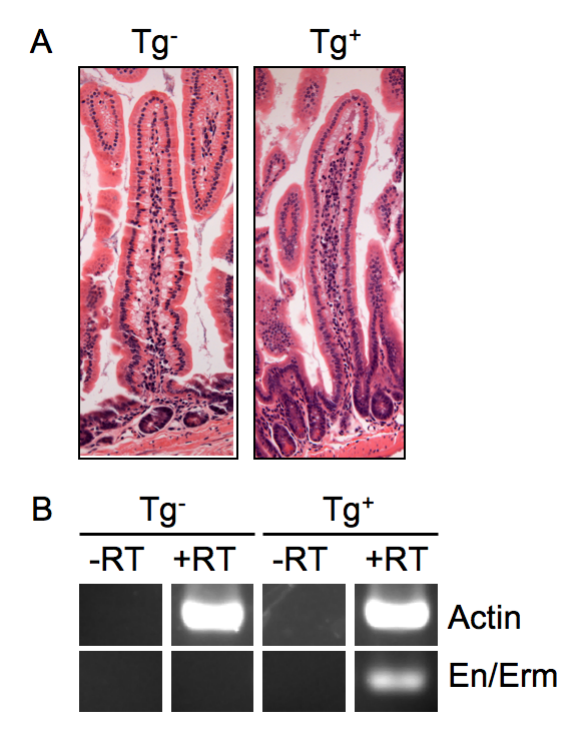
**Intestinal histology and En/Erm expression in transgenic animals in the tumor study**. (A) Representative histology (H+E-stained sections) of mid small intestine from non-transgenic (Tg^-^) and Villin-En/Erm transgenic (Tg^+^) animals under conditions of normal homeostasis. The transgenic animals show crypt-villus morphology indistinguishable from control non-transgenic animals. (B) Levels of Actin and En/Erm transgene RNA in non-transgenic (Tg^-^), and transgenic (Tg^+^) animals, as determined by RT-PCR (cycle number: 25).

We have previously generated and characterized transgenic animals expressing the Ets dominant repressor En/Erm, composed of an HA epitope tag, the *Engrailed *repressor domain and the DNA-binding domain of the Ets factor Erm, in the intestinal epithelium under control of the Villin promoter. En/Erm can potently block transcriptional activation by multiple members of the Ets family and, when expressed at immunohistochemically detectable levels in the intestinal epithelium, causes a severe disturbance of crypt-villus homeostasis [10]. We established additional lines, which stably integrated the Villin-En/Erm transgene, but did not express En/Erm at immunohistochemically detectable levels. Animals from such lines did not manifest an overt disturbance of crypt-villus homeostasis (Figure 1A), indicating a threshold level of transgene expression for the previously described dramatic dysmorphogenesis phenotype [10]. Such animals did, however, express the transgene at levels detectable by RT-PCR (Figure 1B). Expression levels of different Ets factors in the intestine vary widely [9]. We thus wondered whether such low transgene expressors, free of phenotype under conditions of normal homeostasis, might manifest a phenotype under pathologic conditions, by uncovering a differential requirement for endogenous Ets factor(s) expressed at lower relative levels.

We thus examined the effect of low-level En/Erm expression on intestinal epithelial tumorigenesis. Our prior studies showed that the Villin-En/Erm transgene is expressed primarily in the small intestine [10]. We therefore chose the Apc^Min ^mouse as the tumor model, as these animals develop multiple epithelial tumors predominantly in the small intestine [13]. The tumor studies were carried out in a hybrid (C57BL/6J × FVB/N) genetic background, since the Villin-En/Erm transgenic lines were generated in the FVB/N strain while the background of the Apc^Min ^animals was C57BL/6J. As others have done in similar studies [15], we determined the genotype of the major modifier of tumor multiplicity in the Apc^Min ^strain, Mom1, in order to control for possible genetic background differences between control (Apc^Min^) and experimental (Apc^Min^;Villin-En/Erm) groups. Such analyses indicated that all animals in both control and experimental groups were heterozygous for the Mom1 locus, and thus similarly susceptible to Apc^Min^-driven tumorigenesis.

**Figure 2:**
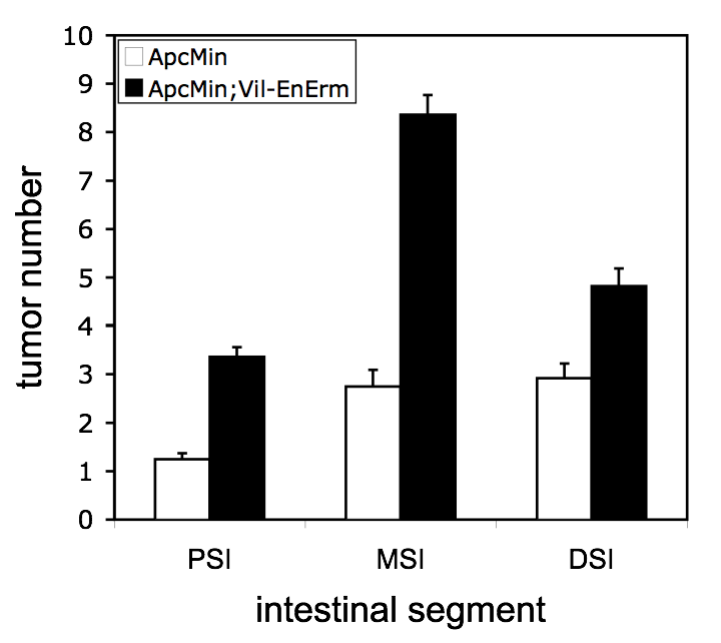
**Tumor number in the small intestines of Apc^Min ^and Apc^Min^; Villin-En/Erm mice, broken down by longitudinal segment (PSI, MSI and DSI denote proximal, mid and distal small intestine, respectively)**. Data are expressed as mean and standard error of the mean.

As shown in Table 1, Apc^Min^;Villin-En/Erm animals developed 2.4 times as many total small intestinal tumors as Apc^Min ^controls (16.5 vs. 6.8, p = 0.03). The Apc^Min^;Villin-En/Erm group included approximately equal numbers of animals from three independent Villin-En/Erm transgenic lines, each of which developed on average more tumors than the Apc^Min ^control group (data not shown), indicating that the differences in tumor number were due to En/Erm expression rather than transgene integration site effects. Apc^Min^;Villin-En/Erm mice developed more tumors in each intestinal segment (proximal, mid and distal) compared to the Apc^Min ^controls, although the differences were greatest and most statistically significant in the mid small intestine (8.4 vs. 2.8, p = 0.03; Table 1 and Figure 2). Interestingly, this is the segment where we previously observed the most severe crypt-villus dysmorphogenesis phenotypes in high En/Erm expressor animals [10]. As expected, tumor numbers in the colon were lower, and not significantly different between the Apc^Min^;Villin-En/Erm and Apc^Min ^groups (0.64 vs. 0.75, p = 0.80), consistent with the lower levels of transgene expression in this part of the intestinal tract [10,18]. There was no significant difference in average overall small intestinal tumor size between the Apc^Min^;Villin-En/Erm and Apc^Min ^groups (Table 1), suggesting that the En/Erm tumor-promoting effect acts predominantly at an early stage of tumorigenesis. One exception was the mid small intestine, where a modest, but statistically significant, increase in tumor size was observed in the Apc^Min^;Villin-En/Erm group (Table 1); hence, locally, En/Erm may also promote later (post-initiation) stages of tumorigenesis. Thus, low-level expression of the Ets dominant repressor En/Erm enhances intestinal tumorigenesis in Apc^Min ^mice.

**Table 1:** Summary of animal tumor data

	Apc^Min^ (n = 12)	Apc^Min^; Villin-En/Erm (n = 11)	
	Mean (SEM)	Mean (SEM)	p-value**

Animal age (months)	14.3 (1.3)	13.0 (1.1)	0.45

Tumor number (total)	6.8 (2.0)	16.5 (3.4)	0.03

PSI	1.3 (0.1)	3.4 (0.2)	0.07

MSI	2.8 (0.3)	8.4 (0.4)	0.03

DSI	2.9 (0.3)	4.8 (0.4)	0.37

Tumor size (total; cm)*	0.31 (0.03)	0.31 (0.02)	0.89

PSI	0.33 (0.07)	0.25 (0.04)	0.33

MSI	0.20 (0.02)	0.27 (0.01)	0.001

DSI	0.32 (0.04)	0.32 (0.02)	0.82

% tumors with stromal invasion	10.9 (4.6)	19.3 (4.6)	0.17

% histologically aggressive tumors	3.7 (2.8)	4.7 (2.4)	0.78

* largest dimension from slide

** from two-tailed student t-test with unequal variance

SEM: standard error of the mean

PSI, MSI and DSI: proximal, mid and distal small intestine, respectively

**Figure 3:**
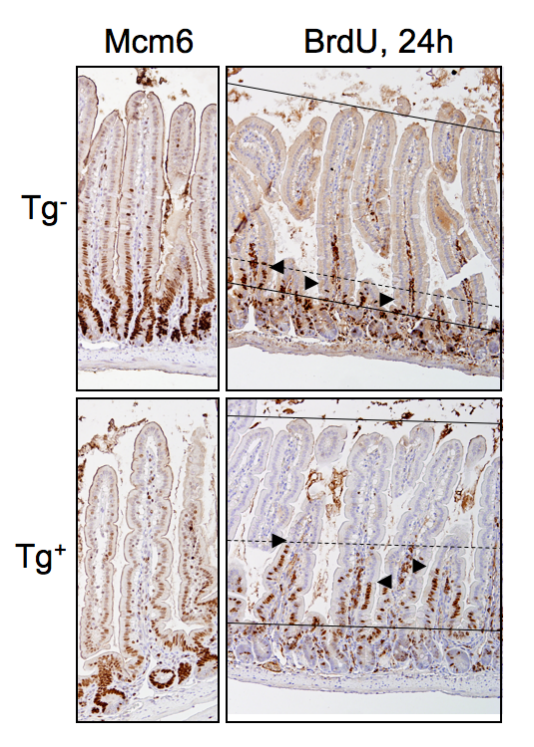
**Analysis of epithelial maturation and transit in the small intestines of En/Erm low-expressor mice (Tg^+^) and non-transgenic controls (Tg^-^)**. Transgenic animals show appropriate loss of nuclear Mcm6 expression along the crypt-villus axis, indistinguishable from controls, reflecting normal epithelial maturation. In contrast, crypt-villus epithelial transit, as determined by in vivo BrdU labeling [10], is increased in transgenic animals compared to controls (top solid line: villus tips; bottom solid line: crypt-villus junction; dashed line: overall upper limit of epithelial transit; arrowheads: upper limit of epithelial transit in individual villi). Representative images of mid small intestine are shown for both groups.

As discussed, the low expressor transgenic animals in the tumor studies did not manifest an overt intestinal dysmorphogenesis phenotype, as judged by H+E histomorphology, in contrast to the previously described high expressor animals [10]. To determine whether more subtle phenotypes might be present under conditions of homeostasis, we performed additional analyses. As shown in Fig. 3, Mcm6 immunohistochemical staining confirmed the presence of normal epithelial maturation in the Villin-En/Erm animals, indistinguishable from non-transgenic controls. Interestingly, by *in vivo *BrdU labeling, the low expressor En/Erm animals did show a modest increase in crypt-villus epithelial transit, smaller than previously described in high expressors [10], but clearly different from non-transgenic controls (Figure 3). Thus, the tumor-promoting effect of En/Erm may act in part by increasing epithelial turnover.

### Effect of En/Erm expression on tumor invasiveness in Apc^Min ^mice

**Figure 4:**
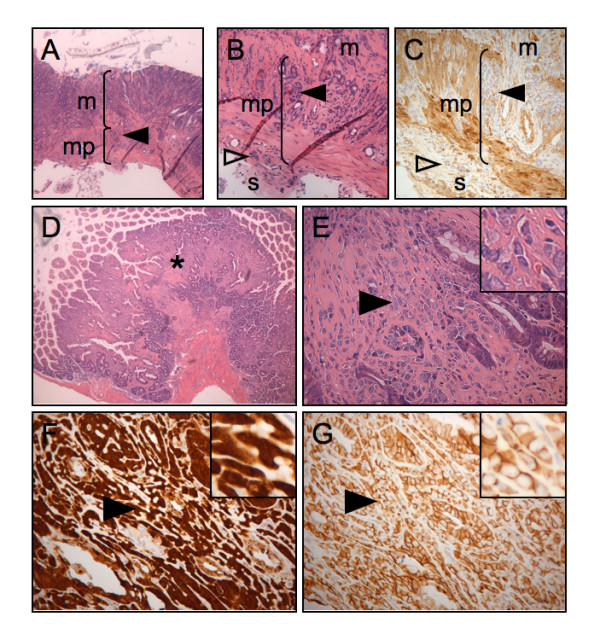
**Invasive adenocarcinomas in animals in tumor study**. (A-C) H+E-stained (A: low-power view; B: high-power view) and SMAimmunostained (C) histologic sections of a representative invasive tumor. Note neoplastic epithelium invading through the muscularis propria (mp) of the intestinal wall (solid arrowheads) and into the outermost serosa layer (s and open arrowheads; m: mucosa; SMA immunostain highlights well-oriented muscularis propria layer). (D-G) Histologically aggressive lesions. H+E-stained (D: lowpower view; E: high-power view) histologic sections showing poorly formed glands and clusters of neoplastic epithelial cells (asterisk in D and arrow in E) in a densely collagenized desmoplastic stroma. These aggressive lesions express high-levels of cytoplasmic and nuclear beta-catenin (arrowhead in F; inset: detailed view), but retain membranous E-cadherin expression (arrowheads in G; inset: detailed view).

By histologic analysis, tumors in both control (Apc^Min^) and experimental (Apc^Min^;Villin-En/Erm) groups included adenomas and adenocarcinomas with similar morphologic features. We found evidence of stromal invasion (of the lamina propria and beyond; Figure 4A–C) in 70% of the animals. There was a trend toward greater invasiveness in the Apc^Min^;Villin-En/Erm group (19.3% vs. 10.9% of tumors), although this difference did not reach statistical significance (p = 0.17) (Table 1). Further, nearly one third of the tumors showing stromal invasion manifested highly aggressive histology, characterized by small, poorly differentiated glands and clusters of malignant epithelial cells in a densely collagenized, desmoplastic stroma (Figure 4D and 4E). Such tumors expressed high levels of both cytoplasmic and nuclear (activated) β-catenin, but retained epithelial characteristics, including membranous E-cadherin expression (Figure 4F and 4G). Adenomas associated with such tumors tended to contain regions with cytologic features of high-grade dysplasia, including large nuclei with prominent nucleoli and brisk mitotic activity (data not shown). The incidence of these aggressive tumors was not significantly different between the experimental (Apc^Min^;Villin-En/Erm) and control (Apc^Min^) groups (4.7% vs. 3.7%, p = 0.78). Thus, a relatively high proportion of Apc^Min^-induced neoplastic lesions gives rise to invasive and histologically aggressive adenocarcinomas in a C57BL/6J × FVB/N hybrid genetic background, and En/Erm-expressing tumors show a trend toward greater stromal invasiveness.

## Discussion

Members of the Ets transcription factor family are frequently misexpressed or otherwise dysregulated in diverse human malignancies [1,4,5]. Although Ets factors have been shown to regulate a variety of cellular processes relevant to tumorigenesis [1,4,5], relatively little is known about their effects on tumor initiation and/or progression *in vivo*. Indeed, to date, few studies have examined Ets functions in tumorigenesis using animal genetic models (Table 2). In mouse models of breast cancer, Pea3 and Ets2 have been shown to exert tumor-promoting effects, the former in the epithelium and the latter in the stroma [6-8]. In the present study, we examined the effect of expression of the Ets dominant repressor transgene En/Erm on Apc^Min^-driven tumorigenesis in the intestine. Suprisingly, we find that low-level expression of En/Erm in the intestinal epithelium more than doubles the tumor number in Apc^Min ^mice. This effect appears to act at an early stage in tumor formation, as En/Erm seems to have little effect on tumor size. Interestingly, while lacking the overt crypt-villus dysmorphogenesis phenotype of high-expressor animals, the low-expressor mice analyzed in this tumor study do retain a degree of increased crypt-villus epithelial transit. This suggests that the En/Erm tumor-promoting effect may act in part through increased epithelial turnover. As an interesting corollary, it also implies that epithelial maturation and transit, found to be coordinately disrupted in the high-expressor En/Erm animals [10], are regulated by different mechanisms, as the phenotypes are genetically separable in the low-expressor mice.

**Table 2:** Summary of genetic studies examining Ets factor functions in tumorigenesis

Ets	Genetic manipulation	Tissue	Tumor model	Effect on tumorigenesis	Subcompartment with effect	Reference
PEA3 subfamily	Dominant negative	Mammary gland	MMTV-Neu	Inhibition (increased latency; decreased number and size)	Epithelium	[7]

Ets2	Hypomorphic mutant	Mammary gland	MMTV-PyMT MMTV-Neu	Inhibition (increased latency; decreased number and size)	Stroma	[6,8]

Ets2	One extra gene copy	Intestine	Apc^Min^	Inhibition (decreased number)	Not determined	[18]

Ets family	Dominant repressor	Intestine	Apc^Min^	Promotion (increased number)	Epithelium	This paper

Most Ets factors function as transcriptional activators [1,2]. Since En/Erm exerts its effect by blocking Ets-mediated transcriptional activation [10], our findings imply that the endogenous Ets factors blocked by En/Erm normally function to restrict tumorigenesis in the intestinal epithelium. Due to the high conservation of the Ets domain, the En/Erm protein is able to block transcriptional activation by multiple different Ets factors [10], making it difficult to determine which individual Ets is/are responsible for this tumorigenesis-modifying effect. We have previously shown that higher (immunohistochemically detectable) levels En/Erm expression result in small intestinal crypt-villus dysmorphogenesis, probably by interfering with the activity of relatively abundant Ets factors in the intestine, such as Elf3, Ehf and/or Ets2 [10]. In contrast, possible candidate Ets factors responsible for the tumor phenotype include those with normally lower relative expression levels in the intestine, such as Pea3, Erm and/or Elf 1 [9]. Alternatively, the tumor phenotype may be uncovering a differential requirement for a more highly expressed Ets. Interestingly, similar to our findings, Sussan et al recently observed an inverse relationship between Ets2 gene copy number and tumor number in the Apc^Min ^model, suggesting that Ets2 normally functions to restrict intestinal tumor formation [19]. Thus, while frequently overexpressed in colon cancer, at least some Ets factors, including Ets2 and those blocked by the En/Erm transgene in our studies, appear to normally restrict, rather than promote, epithelial neoplasia in the intestine. Indeed, it may be that the same Ets factors manifest different functions in neoplasia at different expression levels due to differential promoter binding and regulation.

Secondly, our studies suggest that the Apc^Min ^mutation may provide a good model of human intestinal cancer in the C57BL/6J × FVB/N hybrid genetic background. In this background, the overall tumor burden is lower and the proportion of invasive lesions higher than in the pure C57BL/6J background, in which animals die relatively early from intestinal obstruction caused by numerous non-invasive adenomas. Thus, this mixed background model approximates the human disease, and could be useful for studying genetic parameters controlling carcinoma invasion and metastasis.

## Conclusion

Expression of the Ets dominant repressor En/Erm in the small intestine, at levels that do not cause crypt-villus dysmorphogenesis, results in a marked increase in tumor number in the Apc^Min ^model of intestinal carcinoma. Tumor size is relatively unaffected, indicating that this effect acts predominantly at the level of tumor initiation or/and early promotion. Histologic examination of the tumors suggests that En/Erm expression may also promote stromal invasion. Together, these findings from an animal genetic model provide *in vivo *evidence for an unexpected role for endogenous Ets factors in the restriction of epithelial tumorigenesis in the intestine. Moreover, our studies suggest that the Apc^Min ^mutation may provide a good model for invasive human intestinal carcinoma in the C57BL/6J × FVB/N hybrid genetic background.

## Competing interests

The authors declare that they have no competing interests.

## Authors' contributions

PJ designed the study and analyzed the data. PJ and XS carried out the experiments. PJ and AGH wrote the manuscript. All authors read and approved the final manuscript.

## Pre-publication history

The pre-publication history for this paper can be accessed here:

http://www.biomedcentral.com/1471-2407/9/197/prepub
